# Effect of Couples Counselling on Reported HIV Risk Behaviour among HIV Serodiscordant Couples by ART Use, HIV Status and Gender in Rural Uganda

**DOI:** 10.1371/journal.pone.0136531

**Published:** 2015-09-18

**Authors:** Rachel King, Jeong Min, Josephine Birungi, Maureen Nyonyintono, Katherine A. Muldoon, Sarah Khanakwa, Pontiano Kaleebu, David M. Moore

**Affiliations:** 1 University of California San Francisco, Kampala, Uganda; 2 British Colombia Centre for Excellence in HIV/AIDS, Vancouver, Canada; 3 The AIDS Support Organization, Jinja, Uganda; 4 School of Population and Public Health, University of British Columbia, Vancouver, Canada; 5 Uganda Virus Research Institute/ Medical Research Council Research Unit on AIDS, Entebbe, Uganda; 6 Faculty of Medicine, University of British Colombia, Vancouver, Canada; University of Alabama at Birmingham, UNITED STATES

## Abstract

**Background:**

We examined several measures of self-reported HIV risk behaviour in mutually disclosed sero-discordant couples over time to see if a couples counselling intervention was associated with changes in these behaviors.

**Methods:**

We analysed data from a prospective cohort study of HIV sero-discordant couples in Jinja, Uganda collected between June 2009 and December 2011. Participants received couples counselling, at 3-monthly intervals. We examined trends in reported condom-use, number of concurrent sexual partners, knowledge of HIV serostatus of concurrent partners and condom use of concurrent partners using Generalized Estimating Equation models, comparing responses at study enrollment with responses at six, 12 18 and 24 months of follow-up.

**Results:**

A total of 586 couples were enrolled and the female member was HIV positive in 255 (44%) of them. The median age for female participants was 35 years and 42 years for men. Reported condom use at last sex with spouse increased over time (p<0.001) with the largest increases found among couples where the positive participant never received ART during the study(an increase from 68.8% at enrollment to 97.1% at 24 months). Male participants reported reductions in the number of concurrent sexual partners (p<0.001), increase in the knowledge of the HIV serostatus of these partners (p = 0.001) and a trend towards improved condom-use among non-primary partners (p = 0.070). Reported reduced risky behaviors did not wane over the study period.

**Conclusion:**

Couples counselling resulted in increased condom use among all participants and among men the intervention resulted in reductions in risk behaviour with concurrent sexual partners. Routine counselling for serodiscordant couples should be integrated in routine ART care programs.

## Introduction

HIV serodiscordant couples in stable relationships are a major source of new infections in sub-Saharan Africa.[1–5] Serodiscordance among couples in sub-Saharan Africa is relatively common, occurring in 8–40% of couples who test for HIV representing the single largest group of preventable new infections. [4–7] Cheimatelly summarized DHS data regarding discordant relationships from 20 sub-Saharan African countries and has found that in high-prevalence countries (over 10% HIV prevalence), a large proportion of stable partnerships were affected by HIV and about half were discordant, whereas in low-prevalence countries (less than 10% HIV prevalence), fewer stable partnerships were affected by HIV but a higher proportion of them were discordant. [8] In Uganda, it is estimated that among those infected with HIV and living with a sexual partner, more than 50% of their partners are HIV uninfected and either acquired HIV before meeting their current partner or during a concurrent relationship. [9]While the provision of antiretroviral therapy (ART) has been demonstrated as a highly efficacious intervention to prevent HIV transmission within discordant couples[10], observational studies have shown that the incidence of HIV infection among negative partners of HIV positive individuals receiving ART can still be upwards of 3 per 100 person-years. [11] Among discordant couples who have not initiated ART, incidence can be as high as 25 per 100 person years.[12–16] Consequently, even with the provision of HIV treatment, additional measures are needed for discordant couples to further reduce HIV transmission.

As such, HIV serodiscordant couples, are recognized as a priority for HIV prevention in developing countries.[17] Developing effective HIV prevention interventions that target serodiscordant couples could potentially contribute to reducing HIV transmission in many countries. [1] Previous studies have shown that reported condom use increases after the provision of couples counselling. [18,19] For more than 20 years couples-based HIV counselling and testing (CHCT) is a proven strategy to reduce the risk of HIV transmission between sexual partners.[19–21] [22] However, one study reported that the initial impact of such counselling may wane over time. [23]

We examined several measures of self-reported HIV risk behaviour in men and women over time to assess if couples counselling was associated with changes in different measures of HIV risk behaviour. We examined if the gender of the participant and ART-use play a role in risk behavior. This study was conducted within an observational cohort study of HIV-serodiscordant couples in Jinja, Uganda that was examining the effectiveness of ART as an HIV prevention intervention (TasP).

## Methods

We conducted an analysis from the Highly Active Antiretroviral therapy as Prevention (HAARP) study, a prospective cohort of all HIV sero-discordant couples enrolled from June 2009 to June 2011. Described previously,[7,24,25] the study was comprised of two groups of HIV uninfected individuals, who were co-habiting heterosexual partners of HIV infected individuals receiving HIV care and treatment services from The AIDS Support Organization (TASO) at the Jinja Centre in Eastern Uganda. One group was composed of individuals where the HIV positive partner was receiving ART because of meeting the Ugandan National Clinical or laboratory eligibility requirements, at the time, namely a CD4 cell count ≤250 cells/ μL or WHO Stage III or IV disease. The second group was composed of individuals where the infected partner was not yet ART eligible. If the HIV positive participants subsequently became eligible for ART during the course of the study, they were then started on treatment. The results of the main HAARP study have been reported elsewhere; but in short found that ART-use was not associated with a reduced risk of HIV transmission among serodiscordant couples in this study [25].

All participants were discordant couples and received HIV transmission risk-reduction counselling on a quarterly basis and regular supplies of condoms. HIV incidence in the two groups was measured by testing the uninfected partner for HIV every three months over two years of follow-up. The HAARP study protocol received ethical review and approval by the following ethics bodies: Science and Ethics Committee of the Uganda Virus Research Institute (UVRI), the Research Ethics Board of the University of British Columbia (UBC), and the protocol was registered at the Uganda National Council of Science and Technology (UNCST). All data had no personal identifier information prior to analysis.

Participants were aged 18 years or older and in a co-habiting sexual relationship for at least 6 months. One partner had documented HIV infection and was receiving HIV care from TASO at the Jinja Centre. The other partner was required to have an HIV-negative test during screening for study enrollment. In addition, couples must have been resident in the TASO-Jinja catchment area with intention of continued residence for at least one year and had at least two episodes of reported sexual intercourse in the 3 months prior to screening. Both members of the couple must have been aware of the other’s serostatus prior to enrollment.

After providing written informed consent in a language of their choice (Swahili, Luganda or Lusoga), couples were interviewed by trained research assistants to complete a structured questionnaire which collected basic demographic and socio-economic information, as well as sexual behaviour data. Behavioural data were collected at enrollment and at six month intervals thereafter. Interviews were conducted separately for each member of the couple with a different research assistant and in different rooms in order to limit biased responses due to social desirability. These questionnaires took less than 30 minutes to complete.

On a quarterly basis, each couple was provided with standardized couples counselling for HIV-discordant couples by a study counsellor, based on guidelines developed by TASO and the Ministry of Health in Uganda[26]. Counselling topics included risk reduction methods, regular use of condoms, and educational information about transmission risk between HIV-infected and HIV-uninfected partners. All counselling sessions took place after the behaviour data collection and were conducted by a study staff member who was not involved in the data collection.

We compared responses at baseline between three categories of couples where the HIV positive was: 1) receiving ART at enrollment, 2) not receiving ART at enrollment but started treatment during the study and 3) never receiving ART during the study. Kruskal-Wallis, Chi-square and Fisher’s exact tests were used to test for significant differences. We then conducted tests of trends to determine the effect of the quarterly couples counselling sessions on four self-reported measures of HIV risk behaviour. These measures were: 1) condom use at last sex with primary spousal partner, 2) number of non-spousal or non-primary sex partners in the previous 3 months, 3) knowledge of HIV sero-status of non-spousal or non-primary partners over the last 3 months, and 4) reported condom use with non-primary spouse and non-spousal sex partners. We ran Generalized Estimating Equation (GEE) models for binary data with logit link to account for correlations for repeated measures to test for trends over time. We conducted separate analyses with participants stratified on the basis of HIV serostatus, the gender of the respondent and on the ART status of the HIV positive participant including data gathered from enrollment and each six-monthly visit to a maximum of 24 months of follow-up. All analyses were conducted using SAS version 9.3 (SAS Corporation, Cary NC).

## Results

Of the 620 couples who presented for screening, 586 were found to be eligible and enrolled in the study. A total of 586 couples were enrolled, of which in 255 (44%), the female member was HIV positive. The median age for female participants was 35 years (Inter-quartile range [IQR]: 30–40) and 42 years for men (IQR: 36–48). The majority of couples spoke Lusoga (65%), followed by Luganda (18%) and other languages (17%). Of the 586 couples, 211 (36%) were in polygamous relationships and in 229 (39%) of the couples the male participant was circumcised. In 249 (42%) of the couples, the HIV positive member of the couple was receiving ART at enrollment and these individuals had been receiving ART for a median of 2.3 years (IQR 1.0–3.7 years). Participants were followed for a median of 15.2 months (IQR 6.8–22.3 months) after enrollment.

There were 17 seroconversions in 778 person-years of follow-up among the HIV negative participants for an overall HIV incidence of 2.18 per 100 person-years. As reported in Birungi, 2015[25], there were no differences in HIV incidence by ART-use. Most of these infections were identified at the first or second follow-up visit.

Table 1 shows the analysis comparing variables collected at study enrollment for the couples who were on ART at enrollment; began ART during the study and those who never received ART. We found significant differences in the age of male partners in each of the three groups (median age 40, 41 and 43 years of age for the never on ART, began ART during the study and on ART at enrollment, respectively; p = 0.002). Women in the never on ART group were also younger (median 33 years) than those in couples where the positive participant received ART (median age 36 years for both ART groups; p = 0.002). However, there were no significant differences in the distribution of the HIV positive gender among the three groups.

**Table 1:** Demographic and clinical characteristics reported at enrollment for 586 discordant couples with participants classified on the basis of ART use by the HIV positive participant during the study.

	Not ever on ART during the study	Not on ART at enrollment, but began ART during the study	On ART at enrollment	P- value
	238 (41%)	99 (17%)	249 (42%)	
**Gender of the positive partner** Male	121 (51%)	60 (61%)	150 (60%)	0.074
Female	117 (49%)	39 (39%)	99 (40%)	
**Age of male partner**				
Median (IQR)	40 (34–47)	41 (36–50)	43 (37–50)	0.002
**Age of female partner**				
Median (IQR)	33 (29–40)	36 (30–40)	36 (30–40)	0.002
**Primary Language Spoken (male response) (n = 573)** Lusoga	164 (69%)	63 (65%)	144 (60%)	0.095
Other	72 (31%)	34 (35%)	96 (40%)	
**Circumcision Status of male partner (n = 579)** Yes	102 (43%)	44 (45%)	83 (34%)	0.053
**# of dependent children (male response) Median (IQR)**	7 (5–11)	9 (5–12)	8 (6–12)	0.067
**# of dependent children (female response)**				
**Median (IQR)**	6 (4–9)	7 (5–9)	6 (4–9)	0.506
**Lowest CD4 cell count at enrollment of HIV positive partner (n = 331)**				
Median (IQR)	492 (373–649)	326 (169–393)	135 (50–200)	<0.001
**CD4 cell count at enrollment of HIV positive partner (n = 559)**				
Median (IQR)	515 (389–684)	248 (156–366)	392 (240–539)	<0.001

We found that both male and female participants in couples receiving ART at enrollment were more likely to report always using condoms in the last 3 months (67% for both men and women) in comparison to the never on ART (58% for men; 64% for women) and those who began ART during the study (62% for men; 56% for women; p = 0.003 for men and 0.023 for women) (Table 2). However, we did not find any significant differences in reported condom use at last sex, intergenerational sex, pregnancy intentions or male controlled decision making across the three treatment strata.

**Table 2:** Behavioral characteristics reported at enrollment for 586 discordant couples with participants classified on the basis of ART use by the HIV positive participant during the study.

	Not ever on ART during the study	Not on ART at enrollment, but began ART during the study	On ART at enrollment	P- value
	238 (41%)	99 (17%)	249(42%)	
**Age of sexual debut (male) Median (IQR)**	18 (16–20)	17 (16–19)	18 (16–20)	0.545
**Age of sexual debut (female) Median (IQR)**	16 (15–18)	16 (15–18)	16 (15–18)	0.191
**Condom use in the last 3 months (male)** Always	138 (58%)	61 (62%)	167 (67%)	0.003
Sometimes	51 (21%)	15 (15%)	58 (23%)	
Never	49 (21%)	23 (23%)	24 (10%)	
**Condom use in the last 3 months (female)**				
Always	153 (64%)	54 (56%)	166 (67%)	0.023
Sometimes	40 (17%)	25 (26%)	57 (23%)	
Never	45 (19%)	18 (19)	26 (10%)	
**Intergenerational Sex (>10 yrs age difference between male and female partner)**	83 (35%)	29 (29%)	90 (36%)	0.472
**# of lifetime sex partners (male)** Median (IQR)	6 (4–12)	6 (4–10)	7 (4–13)	0.315
**# of lifetime sex partners (female)** Median (IQR)	3 (2–4)	3 (2–4)	3 (2–4)	0.573
**Polygyneous partnership**	58 (24%)	26 (26%)	61 (25%)	0.928
**Pregnancy Intensions (male) (n = 522)**Yes	88 (41%)	34 (38%)	77 (35%)	0.401
**Pregnancy Intensions (female) (n = 469) Yes**	51 (26%)	25 (30%)	47 (24%)	0.605
**Alcohol use in past 3 months (male)**	92 (39%)	32 (32%)	76 (31%)	0.153
**Alcohol use in past 3 months (female)**	38 (16%)	17 (17%)	36 (15%)	0.796
**Male decides when to use condom (female’s response)**	59 (25%)	19 (19%)	51 (20%)	0.407
**Use Other Family Planning (HIV positive partner’s response)**	29 (12%)	12 (13%)	25 (10%)	0.711
**Used a condom at last sex (%) (males)**	156 (66%)	70 (71%)	187 (75%)	0.069
**Used a condom at last sex (%) (females)**	167 (70%)	67 (68%)	193 (78%)	0.085
**Presence of any non-spousal sex partners in the previous 3 months (male response)**	16 (7%)	7 (7%)	17 (7%)	0.993
**>1 sexual partner reported by female**	4 (2%)	1 (1%)	3 (1%)	0.896
**knowledge of HIV sero-status of non-spousal or non-primary partners over the last 3 months (male)**	43 (64%)	23 (74%)	53(77)	0.246
**knowledge of HIV sero-status of non-spousal or non-primary partners over the last 3 months (female)**	2 (50%)	0 (0%)	1 (33%)	0.999
**Reported condom use with non-primary spouse and non-spousal sex partners (male)**	27 (40%)	13 (42%)	32 (46%)	0.809
**reported condom use with non-primary spouse and non-spousal sex partners (female)**	3 (75%)	0 (0%)	0 (0%)	0.143

### Trends in self-reported HIV risk behaviour associated with couples counselling

#### Tests of Trends in HIV risk behaviour by ART use

Table 3 indicates trends in HIV risk behaviour with participants stratified on the basis of ART status of HIV positive partner. Reported condom use at last sex with spousal partner increased over time across all ART-use strata (p<0.001, for all). For couples where the positive participant was receiving ART at enrollment, condom use increased from 75.5% at enrollment to 92.2% at 12 months and 89.1% at 24 months. For couples where the positive participant began ART during the study, condom-use increased from 68.7% at enrollment to 90.2% at 12 months and 93.3% at 24 months. Condom-use also increased for couples where the positive partner never received ART during the study; from 66.8% at enrollment to 93.4% at 12 months and 97.1% at 24 months.

**Table 3:** Trends in HIV risk behaviour after couples counseling with participants stratified on the basis of ART status of HIV positive partner. Note: N is number of participants who answered the specific questions

			Enrollment	6 months	12 months	18 months	24 months	*p* value
Condom use at last sex with primary partner, as reported by HIV positive participant								
	ART at enrollment	N	249	186	166	127	64	
		N reporting	188	163	153	113	57	
		Percent	75.5%	87.6%	92.2%	89.0%	89.1%	<0.001
	Not on ART at enrollment but started during study	N	99	87	82	57	30	
		N reporting	68	78	74	55	28	
		Percent	68.7%	89.7%	90.2%	96.5%	93.3%	<0.001
	Never on ART	N	238	156	121	80	35	
		N reporting	159	142	113	78	34	
		Percent	66.8%	91.0%	93.4%	97.5%	97.1%	<0.001
Presence of any non-spousal sex partners in the previous 3 months								
	ART at enrollment	N	249	187	166	126	63	
		N reporting	7	5	0	1	1	
		Percent	2.8%	2.7%	0.0%	0.8%	1.6%	0.138
	Not on ART at enrollment but started during study	N	99	87	82	57	30	
		N reporting	4	1	1	1	0	
		Percent	4.0%	1.1%	1.2%	1.8%	0.0%	0.193
	Never on ART	N	238	157	123	81	36	
		N reporting	7	2	1	1	0	
		Percent	2.9%	1.3%	0.8%	1.2%	0.0%	0.139
Knowledge of HIV sero-status of non-spousal or non-primary partners over the last 3 months								
	ART at enrollment	N	38	27	22	17	7	
		N reporting	30	26	20	14	7	
		Percent	78.9%	96.3%	90.9%	82.4%	100.0%	0.369
	Not on ART at enrollment but started during study	N	20	19	12	12	4	
		N reporting	14	15	10	9	4	
		Percent	70.0%	78.9%	83.3%	75.0%	100.0%	0.226
	Never on ART	N	32	18	15	8	8	
		N reporting	20	14	15	7	8	
		Percent	62.5%	77.8%	100.0%	87.5%	100.0%	<0.001
Reported condom use with non-primary spouse and non-spousal sex partners								
	ART at enrollment	N	38	27	22	17	7	
		N reporting	25	23	16	11	6	
		Percent	65.8%	85.2%	72.7%	64.7%	85.7%	0.675
	Not on ART at enrollment but started during study	N	20	19	12	12	4	
		N reporting	9	16	9	9	2	
		Percent	45.0%	84.2%	75.0%	75.0%	50.0%	0.262
	Never on ART	N	32	18	15	8	8	
		N reporting	18	14	12	7	7	
		Percent	56.3%	77.8%	80.0%	87.5%	87.5%	0.040

We did not observe any significant trends across the three ART-use categories with respect to the number of non-spousal or non-primary sex partners in the previous 3 months. However, the number of participants reporting such partners was very low, even at enrollment (2.8%, 4.0% and 2.9%) for the ART-at enrollment group, the started ART during the study group and the “never on ART” group, respectively. Although the proportion of participants reporting such partners declined over time to 1.6%, 0% and 0% at 24 months, respectively, these declines were not statistically significant.

Self-reported knowledge of the HIV-serostatus non-primary sexual partners (predominately co-wives of men in polygynous relationships) was quite high at enrollment (78.9%, 70.0% and 62.5%, across the three groups), but the absolute number of participants with more than one partner was fairly low (30, 14 and 20, respectively). The self-reported knowledge of HIV sero-status for these partners increased to 100% for all three groups by 24 months. However, for only the “never on ART” group were these increases statistically significant (p<0.001).

For the variable condom use with non-primary (or non-spousal) partner, the proportions reporting this behaviour were 65.8%, 45.0% and 56.3%, for each of the three groups at enrollment. These proportions increased for all three groups to 85.7%, 50.0% and 87.5% by 24 months of follow-up. Again only for the “never on ART” group were these increases statistically significant (p = 0.040).

#### Trends in HIV risk behaviour by gender

Table 4 shows the trends in HIV risk behaviour after couples counseling with participants stratified on the basis of gender. Of the 586 HIV-sero-discordant couples enrolled in the HAARP study, reported condom-use at last sex with spousal partner among men increased from 70.5% at enrollment to 91.7% at 12 months and to 92.2% at 24 months follow-up (p<0.001). Among women we observed increases from 72.9% at enrollment to 90.5% at 12 months, to 93.8% at 24 months (p<0.001).

**Table 4:** Trends in HIV risk behaviour after couples counseling with participants stratified on the basis of gender. Note: N is number of participants who answered the specific questions. N reporting is number of participants whose answer is "yes" to the specific questions.

			Enrollment	6 months	12 months	18 months	24 months	*p* value
Condom use at last sex with:	Male	N	586	429	375	258	129	
primary partner, as reported		N reporting	413	385	344	237	119	
by		Percent	70.5%	89.7%	91.7%	91.9%	92.2%	<0.001
	Female	N	586	427	370	269	128	
		N reporting	427	380	335	249	120	
		Percent	72.9%	89.0%	90.5%	92.6%	93.8%	<0.001
Presence of any non-spousal	Male	N	586	431	376	258	132	
sex partners in the previous 3		N reporting	40	16	5	3	2	
months		Percent	6.8%	3.7%	1.3%	1.2%	1.5%	<0.001
	Female	N	586	426	373	269	126	
		N reporting	5	1	5	0	0	
		Percent	0.9%	0.2%	1.3%	0.0%	0.0%	0.267
Knowledge of HIV sero-status	Male	N	167	121	95	64	28	
of non-spousal or non-primary		N reporting	119	106	87	54	26	
partners over the last 3		Percent	71.3%	87.6%	91.6%	84.4%	92.9%	0.001
months*	Female	N	8	4	5	0	1	
		N reporting	3	3	3	0	1	
		Percent	37.5%	75.0%	60.0%	0	100.0%	0.204
Reported condom use with	Male	N	168	121	95	64	28	
non-primary spouse and non-		N reporting	72	68	51	34	18	
spousal sex partners*		Percent	42.9%	56.2%	53.7%	53.1%	64.3%	0.070
	Female	N	8	4	5	0	1	
		N reporting	3	4	2	0	0	
		Percent	37.5%	100.0%	40.0%	0	0	0.714

We found decreases in the proportion of men reporting non-spousal sex partner in the previous 3 month from 6.8% at enrollment to 1.3% to 12 months and 1.5% at 24 months (p<0.001). We did not find significant trends among women with respect to non-spousal sex partners (p = 0.267). However the proportion of women who reported such partners at enrollment were very low (0.9%) and was even lower (0%) by 18 months of follow-up.

Self-reported knowledge of the HIV sero-status of non-spousal or non-primary partners over the last 3 months increased among male participants over time from 71.3% at enrollment to 92.9% at 24 months (p = 0.001). Again, there were no significant trends for this variable among women (p = 0.204) and the absolute number of women reporting non-spousal partners was quite small (maximum of eight participants at enrollment).

Condom-use with non-primary or non-spousal sexual partners increased for men from 42.9% at enrollment to 64.3% at 24 months. However, these increases were only marginally significant (p = 0.070). No significant trends were observed among women (p = 0.714), but again the absolute number of women reporting non-spousal partners was quite small.

#### Tests of Trends in HIV risk behaviour by HIV status

Both HIV positive participants and HIV-negative participants demonstrated increases in condom-use over time (Table 5). Among HIV positive participants condom-use increased from 70.8% at enrollment to 92.2% at 24 months and among HIV negatives the proportion was 72.5% at enrollment to 93.8% at 24 months (p<0.001 for both). Similarly, the proportion of participants reporting non-spousal sexual partners in the previous 3 months declined for both HIV positive participants (3.1% at enrollment to 0.8% at 24 months; p = 0.015) and HIV negative participants (4.6% to 0.8%, respectively; p<0.001). We also found significant increases in the proportion of participants with non-spousal or non-primary partners who knew the serostatus of these partners. Among HIV positive participants this increased from 71.1% at enrollment to 100% at 24 months (p = 0.006) and among HIV negative participants the increase was from 68.2% to 80.0%, respectively (p = 0.024).

**Table 5:** Trends in HIV risk behaviour after couples counseling with participants stratified on the basis of HIV serostatus of the respondent. N reporting is number of participants whose answer is "yes" to the specific questions. Note: N is number of participants who answered the specific questions. As stated in the methods section, we tested for trends over time using Generalized Estimating Equation (GEE) models for binary data with logit link to account for correlations for repeated measures to test for trends over time. All tests were two-sided

			Enrollment	6 months	12 months	18 months	24 months	*p* value
Condom use at last sex	HIV +	N	586	429	369	264	129	
with primary partner,		N reporting	415	383	340	246	119	
as reported by:		Percent	70.8%	89.3%	92.1%	93.2%	92.2%	<0.001
	HIV -	N	586	427	376	263	128	
		N reporting	425	382	339	240	120	
		Percent	72.5%	89.5%	90.2%	91.3%	93.8%	<0.001
Presence of any non-	HIV +	N	586	431	371	264	129	
spousal sex partners in		N reporting	18	8	2	3	1	
the previous 3 months		Percent	3.1%	1.9%	0.5%	1.1%	0.8%	0.015
	HIV -	N	586	426	378	263	129	
		N reporting	27	9	8	0	1	
		Percent	4.6%	2.1%	2.1%	0.0%	0.8%	<0.001
knowledge of HIV	HIV +	N	90	64	49	37	19	
sero-status of non-		N reporting	64	55	45	30	19	
spousal or non-primary		Percent	71.1%	85.9%	91.8%	81.1%	100.0%	0.006
partners over the last 3	HIV -	N	85	61	51	27	10	
months		N reporting	58	54	45	24	8	
		Percent	68.2%	88.5%	88.2%	88.9%	80.0%	0.024
reported condom use	HIV +	N	90	64	49	37	19	
with non-primary		N reporting	52	53	37	27	15	
spouse and non-		Percent	57.8%	82.8%	75.5%	73.0%	78.9%	0.040
spousal sex partners	HIV -	N	86	61	51	27	10	
		N reporting	23	19	16	7	3	
		Percent	26.7%	31.1%	31.4%	25.9%	30.0%	0.949

Lastly, we found increases in condom-use with non-spousal or non-primary sexual partners reported in the previous 3 months among HIV positive participants from 57.8% at enrollment to 78.9% (p = 0.040). For HIV negative participants, the proportion remained largely unchanged from 26.7% at enrollment to 30.0% at 24 months, and this was not statistically significant (p = 0.949).

## Discussion

We demonstrated that a couples counselling intervention among HIV mutually disclosed serodiscordant couples may be effective in increasing the proportion of couples who reported using condoms with primary and secondary sexual partners, reducing the number of sexual partners and increasing the knowledge of participants’ sexual partners serostatus. We would like to note that considering participants in this study were already aware of their partners’ HIV serostatus at enrolment, standard couples counseling at health facilities may have a different, possibly larger effect, if couples were not previously aware that they were in a discordant relationship. Additionally, we’d like to emphasize that, these changes in behavior did not wane over the project period. However, the effect of the counseling was not seen uniformly across all sub-groups when participants were stratified on the basis of gender, HIV serostatus of the respondent or the HIV treatment status of the positive participant. Notably, reported condom-use at enrollment was quite high in this cohort (approximately 70% reported by men) and was highest (75%) for couples where the HIV positive participant was already receiving ART. We believe that the individual HIV risk-reduction counseling that TASO implements as part of its HIV treatment program likely contributed to this effect. There are other programs that provide couples HIV risk reduction counselling that have shown similar results[19,27]. However, even among participants who were not yet receiving ART, reported condom-use was relatively high (approximately 67%), again indicating that TASO interventions for HIV positive individuals not yet receiving ART are also effective at promoting condom use. While this figure does relate to data collected at enrollment in our study, the date of study enrollment was not the date of first contact with TASO, as nearly all of the HIV positive participants had been TASO clients long before the study started. Clearly individuals receiving ART had much more opportunity to be exposed to the TASO counselling services prior to enrollment in the study since they had clinic visits every one to three months. However, most clients who were not receiving ART were also members of TASO and while they were not receiving HIV treatment, most were receiving pre-ART care from TASO in the form of at least quarterly dispensing of cotrimoxazole prophylaxis and at least yearly CD4 cell count monitoring. At some of these visits they may have also been exposed to individual HIV prevention counseling which may explain why their reported condom-use is much higher that what has been reported in the general population in Uganda. Despite the relatively high proportion of individuals reporting condom-use at enrollment, we were able to demonstrate improvements in condom-use across all the strata we examined: men and women, treated and untreated, and HIV-positive and negative participants. We believe that the benefits of couples counseling, and the cost effectiveness compared to biomedical prevention efforts warrant further attention in the HIV prevention paradigm.[22] The effect on reducing the number of secondary sexual partners was only seen in men. This is likely due to the observation that very few women (a maximum of 1.3%) at any time point reported having more than one sexual partner, thus greatly limiting our ability to demonstrate any effect on this outcome. Similarly, we were able to demonstrate an increase in the proportion of men who knew the HIV serostatus of their non-primary sexual partners and to increase condom-use with these partners. This suggests that couples counseling can reduce HIV risk behaviour beyond the couple enrolled in the study, which in this setting where approximately 25% of men are in polygynous relationships and a further 5% of men in single-spouse relationships reported additional sexual partners at enrollment, could be a very important secondary effect. We did not see any waning of this effect over time which contrasts with another study in rural Uganda[23].

While other studies have shown that couples counseling can improve condom-use with the couple dyad participating in the intervention,[27,28] [19–21] [29] we believe that this is the first study to demonstrate reductions in HIV risk behaviour among men with regards to other sexual partners. This is particularly important in our setting where formal multiple partner relationships, such as polygynous marriages are common, and involving all partners in these relationships may be logistically challenging. A previous study in South Africa directed at individuals who attend informal drinking establishments was able to also demonstrate an effect on reducing the number of concurrent sexual partners. [30] Other studies that systematically test partners have shown that awareness of discordance reduces unprotected sex.[27,28,31] The fact that in all categories of ART use, the knowledge of non-primary partner status increased to 100% was remarkable and points to an effect of couples counseling that could lead to increased couple communication and positive health behavior.

Our intervention of couple counselling could be easily added to existing HIV treatment programs with minimal costs. We were able to provide this counseling using two additional counselors for total 586 discordant couples every three months over a two and half year period. In places where TasP may not be feasible or cost-effective, couples counselling has been reported to work even without the addition of TasP[27].

Our study is limited by the self-reported nature of the data we collected and the potential for social-desirability bias. Social-desirability biases would have likely lead to increased reporting of condom-use, a reduction in the reported number of extra-marital sexual partners and an increase in the reported knowledge of the serostatus of non-spousal sexual partners even in the absence of any real change in behaviour. However, among men it is also possible that where up to 30% of men have more than one wife there may have been a social-desirability bias towards reporting an increase number of sexual partners. We do not know if any of the parameters we measured were more, or less sensitive to these biases. However, we attempted to reduce this by ensuring that the data collection and counseling activities were conducted by different groups of staff. As well, we ensured that the behavioural data collection occurred before the counseling sessions at those visits where both activities were conducted.

In conclusion, we found that quarterly couples counseling sessions with serodiscordant couples as part of a study of ART as prevention conducted in a busy ART program in rural Uganda showed increases in reported condom-use for both men and women. As well, we found that among male participants these sessions resulted in reductions in the number of concurrent sexual partners, improvements in the knowledge of the HIV serostatus of these partners and improved condom-use. In this setting, where concurrent sexual partners among the general population are relatively common, widespread implementation of such counseling could result in significant reductions in HIV incidence at a population level.
